# Clinical efficacy of convalescent plasma therapy on treating COVID‐19 patients: Evidence from matched study and a meta‐analysis

**DOI:** 10.1002/ctm2.259

**Published:** 2020-12-21

**Authors:** Weijun Jiang, Weiwei Li, Lei Xiong, Qiuyue Wu, Jian Wu, Bangshun He, Jiawei Shen, Rongrong Pang, Tao Luo, Yanju Guo, Yang Yang, Ying Han, Wei Dai, Peiran Zhu, Xinyi Xia

**Affiliations:** ^1^ COVID‐19 Research Center, Institute of Laboratory Medicine, Jinling Hospital, Nanjing University School of Medicine, The First School of Clinical Medicine Southern Medical University Nanjing China; ^2^ General Clinical Research Center, Nanjing First Hospital Nanjing Medical University Nanjing China; ^3^ Department of Laboratory Medicine Nanjing Red Cross Blood Center Nanjing China; ^4^ Joint Expert Group for COVID‐19 Wuhan Huoshenshan Hospital Wuhan China; ^5^ Department of Laboratory Medicine & Blood Transfusion Wuhan Huoshenshan Hospital Wuhan China

Dear Editor,

Coronavirus disease 2019 (COVID‐19) has infected tens of millions of people worldwide since its pandemic.^1^ No specific therapeutic agents or vaccines for COVID‐19 are available. Convalescent plasma refers to plasma separated from an individual after the infection has subsided and the antibody has developed.^2^, ^3^, ^4^, ^5^ Convalescent plasma therapy (CPT), a classic adaptive immunotherapy, has been successfully used for the prevention and treatment of many infectious diseases for more than one century. Over the past two decades, it was successfully used in the treatment of severe acute respiratory syndrome (SARS) in 2003, H1N1 influenza pandemic in 2009, and Middle East Respiratory Syndrome (MERS) from 2012 to 2017 with satisfactory efficacy and safety. CPT is one of the promising treatment methods and is favored by more and more researchers.^2^, ^3^, ^4^, ^5^, ^6^, ^7^, ^8^, ^9^, ^10^ However, the clinical efficacy and safety of CPT in COVID‐19 remains unclear.

We performed a retrospective observational study by propensity score matching analysis (PSM) and meta‐analysis estimates the clinical efficacy and security of CPT and COVID‐19, which will help inform clinical management of COVID‐19 infection. A total of 326 participants (163 cases group with CPT and 163 matched controls group with the standard treatment) diagnosed as COVID‐19 were included in the clinical study, of which 142 patients (43.56%) were male. The mean age for all patients was 64.07 ± 13.37 years, and the majority (65.64%) of them was more than 60 years old. Of all patients, 79 (48.47%) cases and 77 (47.24%) controls had a history of basic diseases, including hyperlipidemia, diabetes mellitus, coronary heart disease, and tumor.

Interestingly, we found that days of hospital stay in case with CPT groups were significantly higher than matched control group (*P *< 0.0001). Possible explanation is that most of patients in the CPT treatment group condition are relatively serious and nucleic acid test repeated positive. In addition, we also found the rate of male in case with CPT groups were significantly higher than matched control group (*P *< 0.0001). However, no significant differences in age, disease severity, discharge condition, basic disease, and the number in the ICU between case with CPT groups and matched control group were found. The demographic and clinical characteristics of all patients are show in Table S1.

We performed a meta‐analysis of 12 observational studies (1019 cases and 954 controls) that a significant reduction in mortality (odds ratio [OR] = 0.66, 95% confidence interval [CI] = 0.50‐0.86, *P *= 0.002) was found in the CPT group compared with the standard treatment group, and a true positive result was also found in trial sequential analysis (TSA). Our analyses also suggest that CPT may have a clinically relevant impact in reducing the rate of mortality in COVID‐19 (4.91% vs. 9.20%). In subgroup analysis, we revealed significant associations within the Caucasian subgroup (OR = 0.71, 95% CI = 0.52‐0.98, *P *= 0.037), matched study subgroup (OR = 0.49, 95% CI = 0.32‐0.77, *P *= 0.002), severe subgroup (OR = 0.63, 95% CI = 0.40‐1.00, *P *= 0.049), critical subgroup (OR = 0.24, 95% CI = 0.06‐0.92, *P *= 0.037), severity of MIX subgroup (OR = 0.53, 95% CI = 0.33‐0.86, *P *= 0.010), dose of 250‐300 subgroup (OR = 0.41, 95% CI = 0.22‐0.77, *P *= 0.006), no specific dose subgroup (OR = 0.24, 95% CI = 0.06‐0.92, *P *= 0.037), journal of unpublished subgroup (OR = 0.42, 95% CI = 0.26‐0.68, *P *= 0.000), and case size of <50 subgroup (OR = 0.37, 95% CI = 0.21‐0.65, *P *= 0.001), but not the remaining subgroups. In addition, an edge effect may exist in the race of MIX subgroup. Specific data are summarized in Figure 1, Table 1, Figure S1, and Table S2.

**FIGURE 1:**
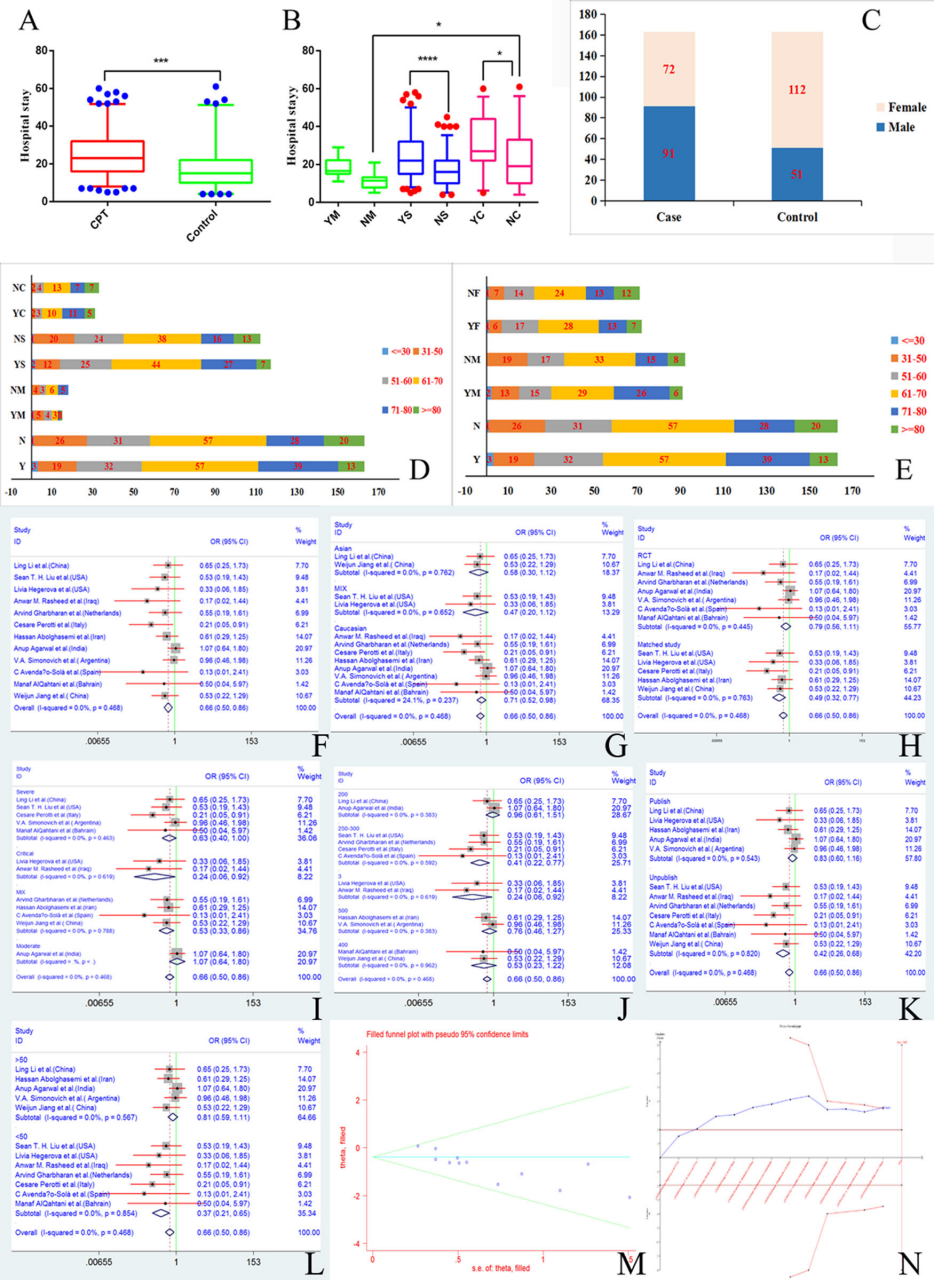
The basic characteristics of all participants and the association between CPT and the rate of mortality. (A‐E) The basic characteristics of all participants. (A) Days of hospital stay in case with CPT groups and matched control group. (B) Days of hospital stay in case and control under severity subgroup. (C) Distribution of male and female in case with CPT groups and matched control group. (D) Age distribution in case with CPT groups and matched control group under severity subgroup. (E) Age distribution in case with CPT groups and matched control group under gender subgroup, *P < .05 (Student's *t*‐test). (F–N) The association between CPT and the rate of mortality. (F) Forest plot for overall analysis. (G) Forest plot for race subgroup analysis. (H) Design subgroup analysis. (I) Forest plot for severity subgroup analysis. (J) Forest plot for dose of CPT subgroup analysis. (K) Forest plot for article states subgroup analysis. (L) Forest plot for case size subgroup analysis. (M) The funnel plot for the association between CPT and the rate of mortality. (N) Trial sequential analysis of CPT and the rate of mortality

**TABLE 1:** Main characteristic of all studies in the meta‐analysis

Author (country)	Race	Study design	Severity	Dose (mL)	Cases/ controls	Journal	Case size	Death case/ control	Discharge case/ control	Improve case/ control	Hospitalization time case/ control	Adverse events case/ control
Li et al ^4^(China)	Asian	RCT	Severe	200	51/50	Publish	>50	8/12	26/18	17/19		2/0
Liu et al^3^ (USA)	MIX	Matched study	Severe	250‐300	39/156	Unpublish	<50	5/38	28/104	32/118		
Hegerova et al^2^ (USA)	MIX	Matched study	Critical		20/20	Publish	<50	2/6	9/9		13.25 ± 1.66/10.25 ± 1.66	0/0
Rasheed et al^9^ (Iraq)	Caucasian	RCT	Critical		21/28	Unpublish	<50	1/8			19.3 ± 6.9/23.64 ± 6.4	1/0
Gharbharan et al^6^ (the Netherlands)	Caucasian	RCT	MIX	250‐300	43/43	Unpublish	<50	6/11				0/0
Perotti et al^8^ (Italy)	Caucasian	Matched study	Severe	250‐300	46/23	Unpublish	<50	3/7				4/0
Hassan et al^5^ (Iran)	Caucasian	Matched study	MIX	500	115/74	Publish	>50	17/18	98/56		9.54 ± 5.07/12.88 ± 7.19	1/0
Agarwal et al^7^ (India)	Caucasian	RCT	Moderate	200	235/229	Publish	>50	34/31			14.25 ± 1.5/13.5 ± 1.3	5/0
Simonovich et al (Argentina)	Caucasian	RCT	Severe	500	228/105	Publish	>50	25/12	171/80			13/2
Avendaño‐Solà et al (Spain)	Caucasian	RCT	MIX	250‐300	38/43	Unpublish	<50	0/4	35/36	35/36		6/7
AlQahtani et al (Bahrain)	Caucasian	RCT	Severe	400	20/20	Unpublish	<50	1/2	19/18			3/0
Jiang et al (China)	Asian	Matched study	MIX	400	163/163	Unpublish	>50	8/15	140/135	151/147	25.02 ± 12.73/17.33 ± 10.94	4/0

We found no evidence of serious adverse events or complications due to CPT, but four participants reported slight transfusion‐related adverse events (clinical symptoms include red, itchy, and inflamed skin) following CPT. TSA found a false positive, although our pooled meta‐analysis found a significant increase in the incidence of adverse events in patients treated with CPT compared to the control group (OR = 2.63, 95% CI = 1.40‐4.94, *P* = 0.003). In subgroup analysis, we revealed significant associations with in the Caucasian subgroup (OR = 2.40, 95% CI = 1.21‐4.79, *P* = 0.013), RCT subgroup (OR = 2.42 95% CI = 1.19‐4.91, *P* = 0.014), severe subgroup (OR = 3.88, 95% CI = 1.24‐12.12, *P* = 0.019), journal of published subgroup (OR = 3.43, 95% CI = 1.23‐9.57, *P* = 0.018), and case size of >50 subgroup (OR = 4.51, 95% CI = 1.58‐12.88, *P* = 0.005), but not the remaining subgroups. In addition, we also found an edge effect in the Asian subgroup, matched study, dose of 200 subgroup, dose of 400 subgroup, and unpublished subgroup. Data on the association between CPT and adverse event risk are summarized in Figure 2, Table 1, Figure S1, and Table S2. No differences between the two groups in terms of hospital stay, improvement of clinical symptoms, and discharge were found (Figures S2‐S5 and Tables S3‐S5).

**FIGURE 2:**
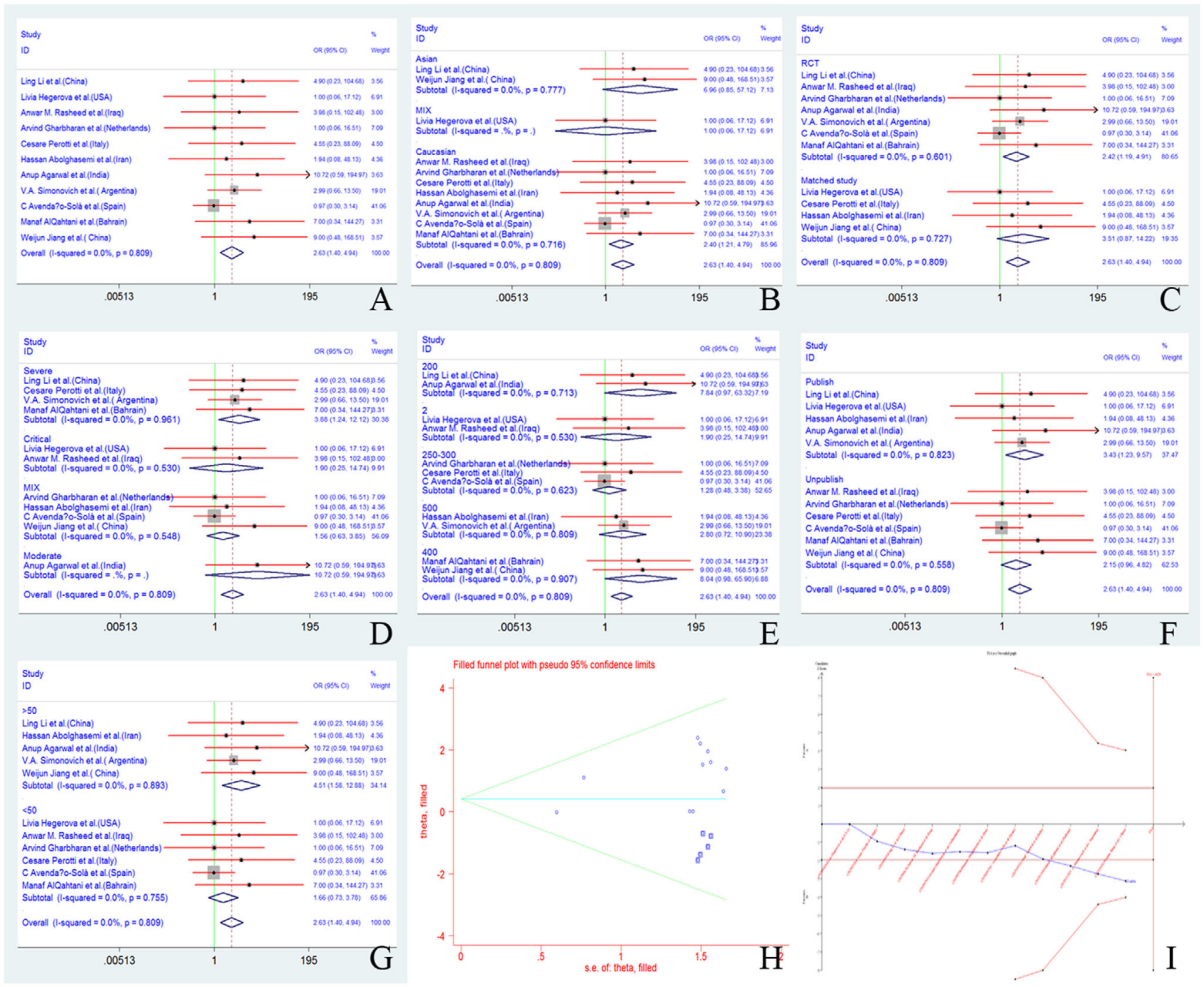
The association between CPT and risk of adverse events. (A‐G) Forest plot for the association between CPT and risk of adverse events. (A) Overall analysis. (B) Race subgroup analysis. (C) Design subgroup analysis. (D) Severity subgroup analysis. (E) Dose of CPT subgroup analysis. (F) Article states subgroup analysis. (G) Case size subgroup analysis. (H) The funnel plot for the association between CPT and risk of adverse events. (I) Trial sequential analysis of CPT and risk of adverse events

Begg's funnel plot and Egger's test were performed to assess publication bias. We additionally conducted sensitivity analyses by omitting one study at a time in the calculation of a summary outcome. Although the sample sizes for cases and controls in all eligible studies varied, corresponding pooled ORs, SMD, and 95%CIs were not qualitatively altered with or without studies on small samples. No other single study influenced pooled OR, SMD, and 95% CI qualitatively.

John Mair‐Jenkins et al^10^ analyzed 32 studies of CPT and SARS coronavirus infection. They revealed consistent evidence for a statistically significant reduction in mortality (OR = 0.25; 95% CI = 0.14‐0.45), especially when convalescent plasma is administered early after symptom onset. However, Li et al,^4^ Agarwal et al^7^, and Simonovich et al reported that CPT was not associated with a reduction in progression to severe COVID‐19 or all‐cause mortality. Our analyses and pooled meta‐analysis suggest that CPT significantly decreased the mortality in COVID‐19 patients.

To the best of our knowledge, it is a systematically review and meta‐analysis of the efficacy and security of CPT to COVID‐19 patients in the largest sample size. We found that CPT significantly decreased the rate of mortality in COVID‐19 patients in our matched control study and meta‐analysis. Our results showed that CPT could significantly reduce the mortality in COVID‐19 patients, and there was no significant increase the incidence of adverse events. These data provide evidence favoring the efficacy and safety of CPT as a therapeutic agent in COVID‐19 patients and provide comprehensive reference for COVID‐19 treatment.

## CONFLICT OF INTEREST

The authors declare that there is no conflict of interest.

## AUTHOR CONTRIBUTIONS

WJJ, XYX, and WWL conceived and designed the experiments. WJJ, QYW, and JW performed publication searches and selection. WJJ, LX, and BSH analyzed the data. JWS, YY, YH, and RRP prepared the figures. WD, TL, PRZ, and YJG contributed materials/analysis tools. WJJ wrote and revised the paper. All authors reviewed the manuscript.

## Supporting information

Supporting Information
