# Supplementary material for: Clinical efficacy of convalescent plasma therapy on treating COVID‐19 patients: Evidence from matched study and a meta‐analysis
Source: Clin Transl Med. 2020 Dec 21;10(8):e259. doi: 10.1002/ctm2.259 (PMC7752155; doi:10.1002/ctm2.259)
Supplement: Supplementary file 1 — Supporting Information [file CTM2-10-e259-s001.docx]

**Clinical efficacy of Convalescent plasma therapy on treating COVID-19 patients：Evidence from matched study and a meta-analysis**

Weijun Jiang^1,#^; Weiwei Li^1,#^; Lei Xiong^1,#^; Qiuyue Wu^1,#^; Jian Wu^1^; Bangshun He^1,2^; Jiawei Shen^1^; Rongrong Pang^1,3^; Tao Luo^1^; Yanju Guo^1^; Yang Yang^1^; Ying Han^1^; Wei Dai^1^; Peiran Zhu^1^; Xinyi Xia^1,4,5*^

**Affiliations**

1COVID-19 Research Center, Institute of Laboratory Medicine, Jinling Hospital, Nanjing University School of Medicine, the First School of Clinical Medicine, Southern Medical University, Nanjing, Jiangsu 210002, China

2General Clinical Research Center, Nanjing First Hospital, Nanjing Medical University, Nanjing, China

3Department of Laboratory Medicine, Nanjing Red Cross Blood Center, Nanjing 210003, Jiangsu, China

4Joint Expert Group for COVID-19, Wuhan Huoshenshan Hospital, Wuhan, Hubei 430100, China

5Department of Laboratory Medicine & Blood Transfusion, Wuhan Huoshenshan Hospital, Wuhan, Hubei 430100, China

**Correspondence**: Xinyi Xia, Prof., M.D. 1. COVID-19 Research Center, Institute of Laboratory Medicine, Jinling Hospital, Nanjing University School of Medicine, the First School of Clinical Medicine, Southern Medical University, Nanjing, Jiangsu 210002, China. 2. Joint Expert Group for COVID-19, Department of Laboratory Medicine & Blood Transfusion, Wuhan Huoshenshan Hospital, Wuhan, Hubei 430100, China E-mail: [xinyixia@nju.edu.cn](mailto:xinyixia@nju.edu.cn)

#Weijun Jiang, Weiwei Li, Lei Xiong and Qiuyue Wu contributed equally to this work and should be considered as joint first authors.


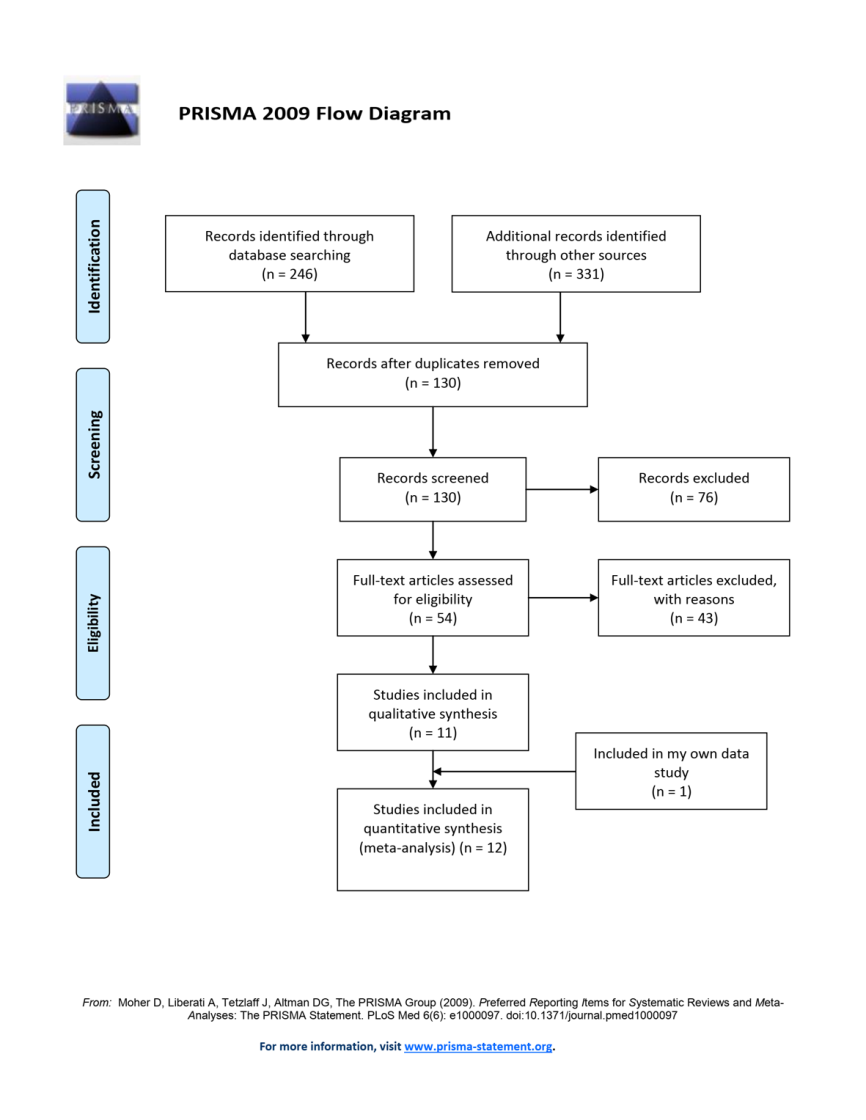


**Supplementary Figure S1 Flow diagram of the study selection process.**

**
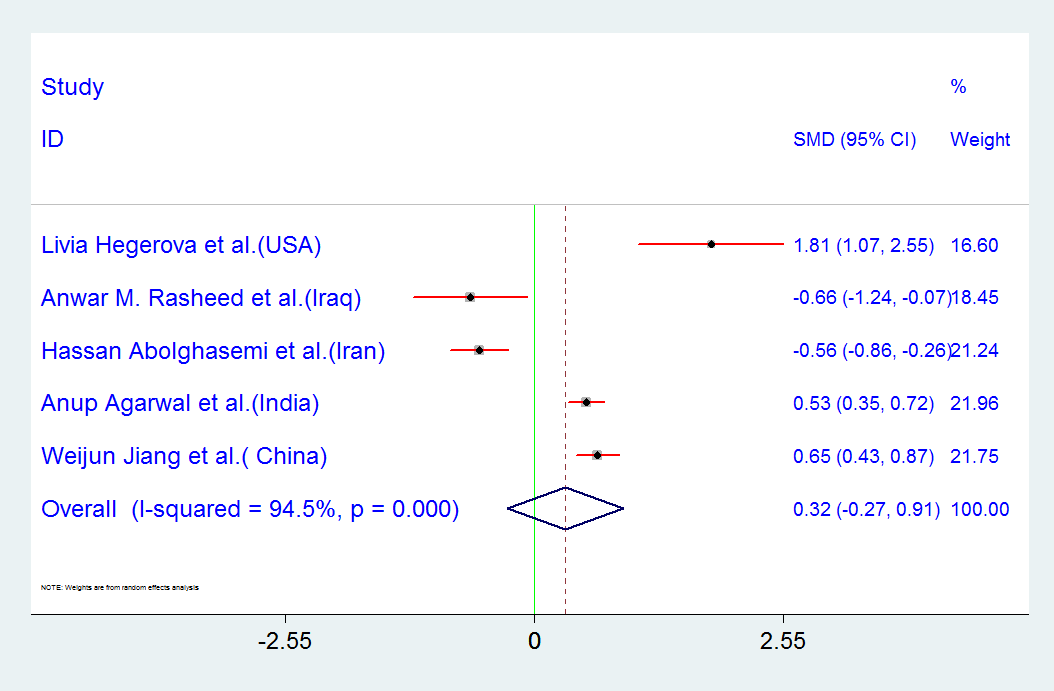
**

**Supplementary Figure S2 Forest plot for the association between CPT and hospital stay in overall analysis.**

**
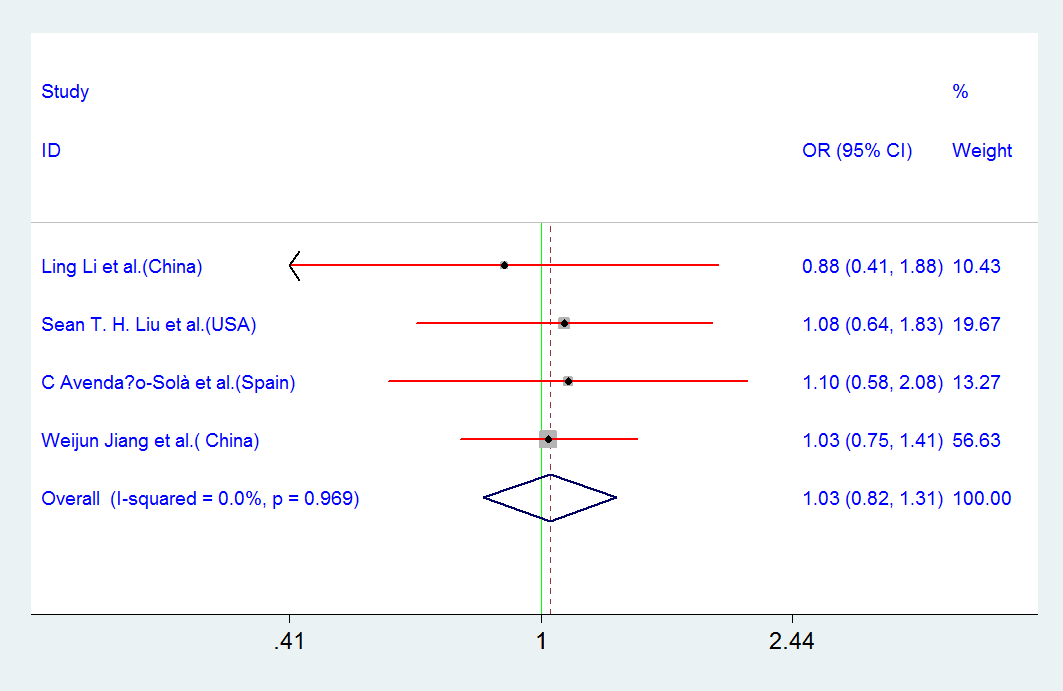
**

**Supplementary Figure S3 Forest plot for the association between CPT and improvement of clinical symptoms in overall analysis**


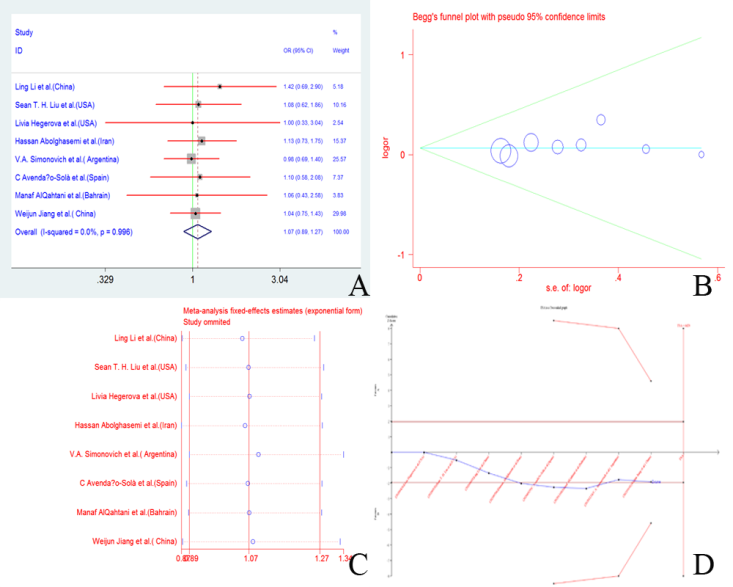


**Supplementary Figure S4 The association between CPT and the rate of discharge (A)** Forest plot for overall analysis **(B)** The funnel plot for the association between CPT and risk of adverse events **(C)** Sensitivity analysis of CPT and risk of adverse events **(D)** Trial sequential analysis of CPT and risk of adverse events


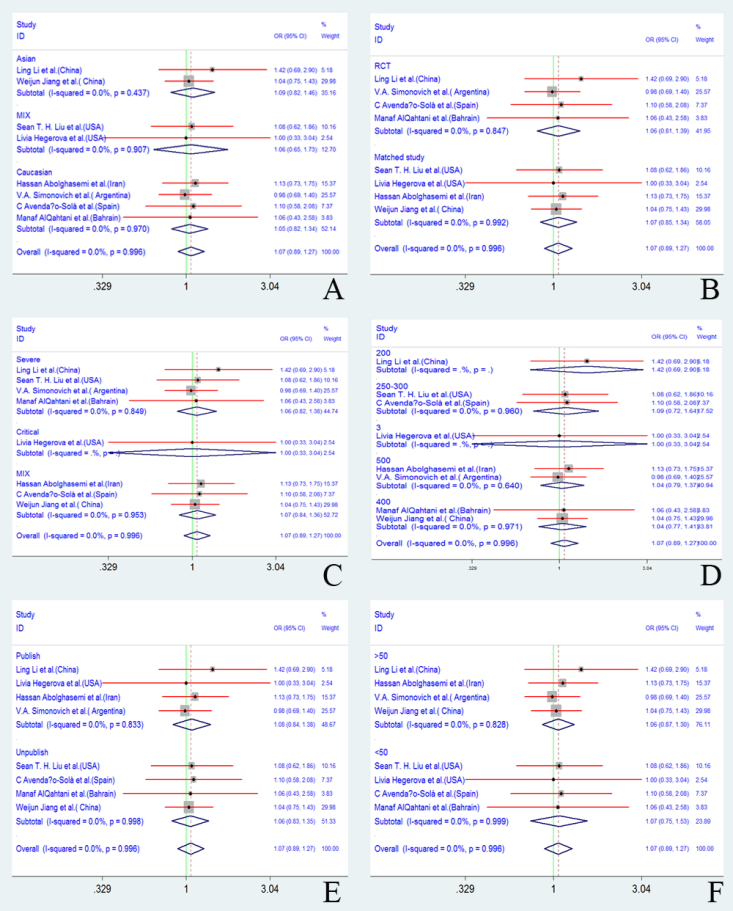


**Supplementary Figure S5 Forest plot for the association between CPT and the rate of discharge.** **(A)** Race subgroup analysis **(B)** Design subgroup analysis **(C)** Case size subgroup analysis **(D)** The article states subgroup analysis

**Supplementary Table S1 Clinical Characteristics of all COVID-19 patients**

| **Characteristics** | **All** | **Case (N=163)** | **Control(N=163)** | ***P* value** |
| --- | --- | --- | --- | --- |
| **Median age (Mean±SD) - yrs** | 64.07±13.37 | 64.22±12.42 | 63.93±14.25 | 0.930 |
| **Hospital stay (IQR)-ds** | 19(11-26) | 23(16-32) | 15(10-22) | **<0.0001** |
| **ICU No. (%)** |  |  |  | 0.755 |
| No | 278(85.28%) | 140(85.89%) | 138(84.66%) |  |
| Yeas | 48(14.72%) | 23(14.11%) | 25(15.34%) |  |
| **Gender group No. (%)** |  |  |  | **<0.0001** |
| Male | 142（43.56%） | 91（55.83%） | 51（31.29%） |  |
| female | 184（56.44%） | 72（44.17%） | 112（68.71%） |  |
| **Basic disease No. (%)** |  |  |  | 0.851 |
| No | 171(52.45%) | 85(52.15%) | 86(52.76%) |  |
| Only one | 99(30.37%) | 49(30.06%) | 50(30.67%) |  |
| Two and More than Two | 56(17.18%) | 29(17.79%) | 27(16.56%) |  |
| **Discharge conditions No. (%)** |  |  |  | 0.255 |
| Cure | 275(84.36%) | 140(85.89%) | 135(82.82%) |  |
| Improve | 23(7.06%) | 11(6.75%) | 12(7.36%) |  |
| death | 23(7.06%) | 8(4.91%) | 15(9.20%) |  |
| Transfer to another hospital | 5(1.53) | 4(2.45%) | 1(0.62%) |  |
| **Disease Severity No. (%)** |  |  |  | 0.801 |
| Moderate | 33(7.14%) | 15(9.20%) | 18(11.04%) |  |
| Severe | 229(9.09%) | 117(71.78%) | 112(68.71%) |  |
| Critical | 64(106.25%) | 31(19.02%) | 33(20.25%) |  |

**Supplementary Table S2 the association between CPT and the rate of mortality and adverse events**

| **Mortality** | **OR (95% CI)** | ***P*** | ***P_h_*** | ***I*^2^%** | ***P*_b_** | **Adverse events** | **OR (95% CI)** | ***P*** | ***P_h_*** | ***I*^2^%** | ***P*_b_** |
| --- | --- | --- | --- | --- | --- | --- | --- | --- | --- | --- | --- |
| **Total** | **0.66(0.50-0.86)** | **0.002** | **0.468** | **0.0%** | 0.001 | **Total** | **2.63(1.40-4.94)** | **0.003** | **0.809** | **0.0%** | 0.050 |
| **Race** |  |  |  |  |  | **Race** |  |  |  |  |  |
| Asian | 0.58(0.30-1.12) | 0.107 | 0.762 | 0.0% |  | Asian | *6.96(0.85-57.12)* | *0.071* | *0.777* | *0.0%* |  |
| MIX | *0.47(0.20-1.12)* | *0.088* | *0.652* | *0.0%* |  | MIX | 1.000(0.06-17.12) | 1.000 | - | - |  |
| Caucasian | **0.71(0.52-0.98)** | **0.037** | **0.237** | **24.1%** |  | Caucasian | **2.40(1.21-4.79)** | **0.013** | **0.716** | **0.0%** |  |
| **Design** |  |  |  |  |  | **Design** |  |  |  |  |  |
| RCT | 0.79(0.56-1.11) | 0.171 | 0.445 | 0.0% |  | RCT | **2.42(1.19-4.91)** | **0.014** | **0.601** | **0.0%** |  |
| Matched study | **0.49 (0.32-0.77)** | **0.002** | **0.763** | **0.0%** |  | Matched study | *3.51 (0.87-14.22)* | *0.078* | *0.727* | *0.0%* |  |
| **Severity** |  |  |  |  |  | **Severity** |  |  |  |  |  |
| Severe | **0.63(0.40-1.00)** | **0.049** | **0.463** | **0.0%** |  | Severe | **3.88(1.24-12.12)** | **0.019** | **0.961** | **0.0%** |  |
| Critical | **0.24(0.06-0.92)** | **0.037** | **0.619** | **0.0%** |  | Critical | 1.90(0.25-14.74) | 0.539 | 0.530 | 0.0% |  |
| MIX | **0.53(0.33-0.86)** | **0.010** | **0.788** | **0.0%** |  | MIX | 1.56(0.63-3.85) | 0.334 | 0.548 | 0.0% |  |
| Moderate | 1.07(0.64-1.80) | 0.802 | - | - |  | Moderate | 10.72(0.59-194.97) | 0.109 | - | - |  |
| **Dose** |  |  |  |  |  | **Dose** |  |  |  |  |  |
| 200 | 0.96(0.61-1.51) | 0.851 | 0.383 | 0.0% |  | 200 | *7.84(0.97-63.32)* | *0.053* | *0.713* | *0.0%* |  |
| 250-300 | **0.41(0.22-0.77)** | **0.06** | **0.592** | **0.0%** |  | N/A | 1.90(0.25-14.74) | 0.539 | 0.530 | 0.0% |  |
| N/A | **0.24(0.06-0.92)** | **0.037** | **0.619** | **0.0%** |  | 250-300 | 1.28(0.48-3.38) | 0.619 | 0.623 | 0.0% |  |
| 500 | 0.76(0.46-1.27) | 0.299 | 0.383 | 0.0% |  | 500 | 2.80(0.72-10.90) | 0.139 | 0.809 | 0.0% |  |
| 400 | 0.53(0.23-1.22) | 0.135 | 0.962 | 0.0% |  | 400 | *8.04(0.98-65.90)* | *0.052* | *0.907* | *0.0%* |  |
| **Journal** |  |  |  |  |  | **Journal** |  |  |  |  |  |
| Publish | 0.83(0.60-1.16) | 0.276 | 0.543 | 0.0% |  | Publish | **3.43(1.23-9.57)** | **0.018** | **0.823** | **0.0%** |  |
| Unpublish | **0.42(0.26-0.68)** | **0.000** | **0.820** | **0.0%** |  | Unpublish | *2.15(0.96-4.82)* | *0.062* | *0.558* | *0.0%* |  |
| **Case size** |  |  |  |  |  | **Case size** |  |  |  |  |  |
| >50 | 0.81(0.59-1.11) | 0.196 | 0.567 | 0.0% |  | >50 | **4.51(1.58-12.88)** | **0.005** | **0.893** | **0.0%** |  |
| <50 | **0.37(0.21-0.65)** | **0.001** | **0.854** | **0.0%** |  | <50 | 1.66(0.73-3.78) | 0.227 | 0.755 | 0.0% |  |

RCT, Randomized Clinical Trial; OR, odds ratio; CI, confidence interval. *P*_h_, *P* value of heterogeneity. *P* value of Q-test for the heterogeneity test. *I*^2^: 0–25, no heterogeneity; 25–50, modest heterogeneity; 50, high heterogeneity. Bold font mean statistically significant results. Italics font mean a possible edge effect

**Supplementary Table S3 Association between length of hospital stay and CPT**

|  | **SMD (95% CI)** | ***P*** | ***P_h_*** | ***I*^2^%** | ***P*_b_** |
| --- | --- | --- | --- | --- | --- |
| **Total** | 0.32(-0.27-0.91) | 0.291 | 0.000 | 94.5% | 0.151 |
| **Race** |  |  |  |  |  |
| Asian | **0.65(0.43-0.87)** | **0.000** | - | - |  |
| MIX | **1.81(1.07-2.55)** | **0.000** | - | - |  |
| Caucasian | -0.21(-1.07—0.67) | 0.643 | 0.000 | 95.6% |  |
| **Design** |  |  |  |  |  |
| RCT | -0.03(-1.85—2.98) | 0.647 | 0.000 | 93.2% |  |
| Matched study | 0.583(-0.49-1.65) | 0.286 | 0.000 | 96.5% |  |
| **Severity** |  |  |  |  |  |
| Critical | 0.56(-1.85-2.98) | 0.647 | 0.000 | 96.2% |  |
| MIX | 0.05(-1.13-1.23) | 0.935 | 0.000 | 97.5% |  |
| Moderate | **0.53(0.35-0.72)** | **0.000** | - | - |  |
| **Dose** |  |  |  |  |  |
| N/A | 0.56(-1.85-2.98) | 0.647 | 0.000 | 96.2% |  |
| 500 | **-0.56(-0.86—0.26)** | **0.000** | - | - |  |
| 200 | **0.53(0.35-0.72)** | **0.000** | - | - |  |
| 400 | **0.65(0.43-0.87)** | **0.000** | - | - |  |
| **Journal** |  |  |  |  |  |
| Publish | 0.53(-0.45—1.52) | 0.289 | 0.000 | 96.3% |  |
| Unpublish | 0.02(-1.25-1.30) | 0.970 | 0.000 | 94.1% |  |
| **Case size** |  |  |  |  |  |
| >50 | 0.22(-0.42-0.86) | 0.506 | 0.000 | 95.7% |  |
| <50 | 0.57(-1.85-2.98) | 0.647 | 0.000 | 96.2% |  |

RCT, Randomized Clinical Trial; OR, odds ratio; CI, confidence interval. *P*_h_, *P* value of heterogeneity. *P* value of Q-test for the heterogeneity test. *I*^2^: 0–25, no heterogeneity; 25–50, modest heterogeneity; 50, high heterogeneity. Bold font mean statistically significant results. Italics font mean a possible edge effect

**Supplementary Table S4 Association between the rate of discharge and CPT**

|  | **OR (95% CI)** | ***P*** | ***P_h_*** | ***I*^2^%** | ***P*_b_** |
| --- | --- | --- | --- | --- | --- |
| **Total** | 1.07(0.89-1.27) | 0.477 | 0.996 | 0.0% | 0.285 |
| **Race** |  |  |  |  |  |
| Asian | 1.09(0.82-1.46) | 0.552 | 0.437 | 0.0% |  |
| MIX | 1.06(0.65-1.73) | 0.811 | 0.907 | 0.0% |  |
| Caucasian | 1.05(0.82-1.34) | 0.707 | 0.970 | 0.0% |  |
| **Design** |  |  |  |  |  |
| RCT | 1.06(0.81-1.39) | 0.649 | 0.847 | 0.0% |  |
| Matched study | 1.07(0.85-1.34) | 0.585 | 0.992 | 0.0% |  |
| **Severity** |  |  |  |  |  |
| Severe | 1.06(0.82-1.38) | 0.654 | 0.849 | 0.0% |  |
| Critical | 1.00(0.33-3.04) | 1.000 | - | - |  |
| MIX | 1.07(0.84-1.27) | 0.571 | 0.953 | 0.0% |  |
| **Dose** |  |  |  |  |  |
| 200 | 1.42(0.69-2.90) | 0.341 | - | - |  |
| 250-300 | 1.09(0.72-1.64) | 0.694 | 0.960 | 0.0% |  |
| N/A | 1.00(0.33-3.04) | 1.000 | - | - |  |
| 500 | 1.04(0.79-1.37) | 0.792 | 0.640 | 0.0% |  |
| 400 | 1.04(0.77-1.41) | 0.803 | 0.971 | 0.0% |  |
| **Journal** |  |  |  |  |  |
| Publish | 1.08(0.84-1.38) | 0.566 | 0.833 | 0.0% |  |
| Unpublish | 1.06(0.83-1.35) | 0.665 | 0.998 | 0.0% |  |
| **Case size** |  |  |  |  |  |
| >50 | 1.06(0.87-1.30) | 0.549 | 0.828 | 0.0% |  |
| <50 | 1.07(0.75-1.53) | 0.700 | 0.999 | 0.0% |  |

RCT, Randomized Clinical Trial; OR, odds ratio; CI, confidence interval. *P*_h_, *P* value of heterogeneity. *P* value of Q-test for the heterogeneity test. *I*^2^: 0–25, no heterogeneity; 25–50, modest heterogeneity; 50, high heterogeneity. Bold font mean statistically significant results

**Supplementary Table S5 Association between clinical symptom improvement rate and CPT**

|  | **OR (95% CI)** | ***P*** | ***P_h_*** | ***I*^2^%** | ***P*_b_** |
| --- | --- | --- | --- | --- | --- |
| **Total** | 1.03(0.82-1.31) | 0.790 | 0.969 | 0.0% | 0.782 |
| **Race** |  |  |  |  |  |
| Asian | 1.00(0.75-1.34) | 0.979 | 0.707 | 0.0% |  |
| MIX | 1.08(0.64-1.83) | 0.761 | - | - |  |
| Caucasian | 1.10(0.58-2.08) | 0.769 | - | - |  |
| **Design** |  |  |  |  |  |
| RCT | 1.00(0.61-1.63) | 0.993 | 0.655 | 0.0% |  |
| Matched study | 1.04(0.80-1.36) | 0.765 | 0.861 | 0.0% |  |
| **Severity** |  |  |  |  |  |
| Severe | 1.01(0.66-1.56) | 0.954 | 0.653 | 0.0% |  |
| MIX | 1.04(0.79-1.38) | 0.780 | 0.850 | 0.0% |  |
| **Dose** |  |  |  |  |  |
| 200 | 0.88(0.41-1.88) | 0.736 | - | - |  |
| 250-300 | 1.09(0.73-1.64) | 0.674 | 0.973 | 0.0% |  |
| 400 | 1.03(0.75-1.41) | 0.867 | - | - |  |
| **Journal** |  |  |  |  |  |
| Publish | 0.88(0.41-1.88) | 0.736 | - | - |  |
| Unpublish | 1.05(0.82-1.35) | 0.697 | 0.973 | 0.0% |  |
| **Case size** |  |  |  |  |  |
| >50 | 1.00(0.75-1.34) | 0.979 | 0.707 | 0.0% |  |
| <50 | 1.09(0.73-1.64) | 0.674 | 0.973 | 0.0% |  |

RCT, Randomized Clinical Trial; OR, odds ratio; CI, confidence interval. *P*_h_, *P* value of heterogeneity. *P* value of Q-test for the heterogeneity test. *I*^2^: 0–25, no heterogeneity; 25–50, modest heterogeneity; 50, high heterogeneity. Bold font mean statistically significant results
